# Did you really need to ask? Cultural variation in emotional responses to providing solicited social support

**DOI:** 10.1371/journal.pone.0219478

**Published:** 2019-07-12

**Authors:** Kendall A. Lawley, Zachary Z. Willett, Christie N. Scollon, Barbara J. Lehman

**Affiliations:** Department of Psychology, Western Washington University, Bellingham, Washington, United States of America; Università degli Studi di Perugia, ITALY

## Abstract

Most prior research on culture and the dynamics of social support has focused on the emotional outcomes for social support recipients. Though an existing body of research has identified cross-cultural differences in the emotional correlates of receiving different types of social support, researchers have seldom examined possible cultural differences in the experience of social support *providers*. This study used the Day Reconstruction Method to examine cultural differences in the emotional correlates of the provision of solicited and unsolicited and emotional and informational social support in the daily lives of Singaporean (n = 79) and American (n = 88) participants. Singaporean participants reported providing more social support overall. Regardless of culture, participants reported more positive emotion (affection, happiness) and less negative emotion (anger, anxiety) when they provided emotional social support. Also, multilevel modeling analyses revealed a 3-way interaction between culture, social support provision, and social support solicitation, indicating cultural differences in negative emotional responses to providing solicited social support. Specifically, results suggest that attempts to provide more solicited social support were associated with more negative emotions in the U.S. In contrast, provider negative emotions were highest in Singapore when the provider did not meet the recipient’s request for support. Patterns of cultural differences in social support provision are dissimilar to—rather than simply mirroring—those found in published research on social support receipt, highlighting the importance of studying social support provision as a distinct phenomenon.

## Introduction

Cultural beliefs and practices can influence how provision of social support (SS), in its many forms, affects SS providers. While cultural differences in the effects of SS *receipt* have been studied in recent years [1–5], the interaction between the dynamics of SS *provision* and culture has been left largely unexamined. Notable exceptions include investigations into SS provision [1, 6]. In the present study, we investigated which, if any, of the recognized cross-cultural differences in SS receipt have parallels in SS provision, and how SS provision and culture interact. To accomplish this, it is necessary to first define SS, and to outline the ways in which SS receipt varies by culture.

### Social support

Social support is critically important for health and well-being throughout the entire lifespan [7]. SS can come in practical or problem-focused forms including material or informational aid, or through emotional means, which make the recipient feel understood, cared for, and part of a mutually beneficial social network [8]. In addition to its emotional benefits, SS is also one of the most widely recognized buffers of physical illness and mortality [9]. Previous research indicates that individuals who receive very little SS, or who are socially isolated suffer a 50% to 91% greater risk of mortality, a factor that predicts mortality about as well as alcohol consumption and smoking [10]. For example, Yang and colleagues found that ratings of feelings of social connectedness predicted lower systolic blood pressure, body mass index, waist circumference, and C-reactive protein levels, all of which are well established biomarkers of physiological health [11]. Although the need for SS appears to be universal, the nuances of SS exchange, such as the type, frequency, intention of the provider, and identity of the recipient can all be colored by cultural context [12].

### Cultural differences in SS exchange

Culture is the system of meanings, folk beliefs, values, practices, and customs of a group of people. Living within a culture necessarily involves repeated engagement in culturally specific behaviors and cognitions, a pattern that affects psychology at every level, from social interaction to neuroanatomy [13]. Culture has also been shown to influence both SS-seeking and SS-providing behavior [6,7]. In particular, prior research suggests that cultural values and SS norms can influence the perceived appropriateness and ratings of effectiveness of various forms of SS.

Perhaps the most commonly studied dimension on which cultures are compared along is the independence-interdependence continuum. This dimension captures the degree of importance that relationships and group membership play in one's self-construal, or view of one’s self. In more interdependent contexts, individuals are more likely to think of themselves in terms of their role in important relationships (“I am Michael’s sister”), or in terms of group identity (“I am an American.” [14]). In more independent cultures, the individual is seen as more autonomous and the self as less “embedded” within relationships and the larger society. Values such as individualism and autonomy are emphasized, and the needs of the individual may be prioritized over those of the group. In independent cultures, social schemas in which the self serves as the primary referent of thought, action, and emotion are more dominant [15]. Independence is characteristic of modern Western cultures such as the United States, whereas interdependence is commonly associated with East Asian cultures like Japan and China. Interdependent cultures emphasize values like communalism, cooperation, and collectivism. Consequently, those in more interdependent cultures generally place greater emphasis on the maintenance of group harmony and prioritize problems of the group. The present study compares the relationship between specific emotions and SS provision in the highly independent culture of the United States and the relatively more interdependent culture of Singapore [16]. We expect that differences in culture will promote differences in the styles of SS that participants from each culture will provide. Although the primary focus of this study is to compare the dynamics of SS provision with those of SS receipt rather than to study cultural differences or speculate upon what mechanisms drive them, we suggest that differences in our Singaporean and American sample are, by definition, cultural differences that can be partially explained by the two cultures’ differences along the interdependence-independence continuum.

### Emotion versus problem-focused support

Social support is often categorized as either emotion- or problem-focused. Problem-focused support refers to SS that is intended to help the recipient eliminate the stressor [17]. Typically, problem-focused SS is provided by sharing information that helps resolve the stressor (advice-giving), or by providing instrumental assistance such as giving a ride to the airport or loaning money. In contrast, emotion-focused support refers to SS intended to assist the recipient in coping with the negative emotions and stress caused by the stressor. Emotion-focused SS includes supportive acts such as comforting, expressing affection and providing encouragement. Research on the relationship between SS and culture suggests that in more interdependent cultures, SS exchanges tend to be more problem-focused, that is, support that is more aimed at addressing the stressor itself. In contrast, SS exchange in more independent cultural contexts tends to focus on addressing recipients’ emotional needs through approaches such as comforting and esteem-boosting [1,7]. In interdependent cultures, requiring emotional SS can be seen as disruptive to group cohesion, but problem-focused SS is not. In independent cultures, emotional SS can be used to bolster self-esteem, which is seen as an important trait in independent cultural contexts. In contrast, problem-focused SS in independent cultures can undermine an individual’s sense of self-efficacy.

### Solicited versus unsolicited support

One important characteristic of SS provision is whether or not the recipient asked for support. Taylor, Sherman, Kim, Jarcho, Takagi, & Dunagan found that Asians and Asian Americans requested less SS than their European American counterparts [4]. This finding was especially true of requests for emotion-focused SS. Taylor and colleagues suggested that Asians and Asian Americans may request less SS due to fear of straining relationships, feeling burdensome, loss of dignity, or disrupting group harmony. Additionally, Mojaverian and Kim found that Asian Americans reported more positive outcomes, such as higher self-esteem and less stress, when receiving unsolicited support than solicited support, whereas there was no difference in outcomes for European Americans when comparing receipt of solicited SS to receipt of unsolicited support [3]. Further, Taylor, Welch, Kim, and Sherman found that SS recipients from more interdependent contexts reported greater stress and negative emotions when support was requested than when they received unsolicited support [5]. Taken together, this research suggests that within interdependent cultures, it is less common and potentially less beneficial to receive SS that has been explicitly requested.

### Receipt versus provision

Much of the research we have reviewed on cultural differences in SS exchange has emerged from research focused on SS recipients. In some cases, these findings provide clear insight into how SS provision may vary across cultures. For example, research indicates that within highly interdependent cultures, a greater proportion of SS receipt is unsolicited [5]. It stands to reason then that within the same culture, a greater proportion of SS provision must be unsolicited as well. However, the extent to which other SS receipt findings generalize to SS provision is less clear. For instance, researchers have found that within highly interdependent cultures, SS recipients are more likely to endorse feelings of burdensomeness and shame when they require or when they receive SS [18,19]. In this case, it is not obvious how recipients’ feelings of burdensomeness and shame affect SS providers. They may mirror the negative emotions of recipients (i.e., feel that their recipients are an irritating burden and/or feel shame on their behalf), or the opposite could be true (i.e., they may feel especially pleased to be of service to their partners). It is also possible that cultural characteristics that lead SS recipients to feel anxious about burdening their support providers may have little effect on providers. To examine whether interactions between culture and SS receipt are paralleled in interactions of culture and SS *provision*, we have selected two variables along which cross-cultural differences in SS exchange have been observed and supported. Specifically, we examine the specific emotional correlates of SS providers who provided both solicited and unsolicited emotional SS and informational SS, a common form of problem-focused SS.

### Culture and specific emotions

Both between-culture and within-culture factors contribute to the experience and expression of emotions. Kuppens, Ceulemans, Timmerman, Diener, and Kim-Prieto refer to these cultural factors that contribute to the experience and expression of emotions as dimensions of emotional experience [20]. They describe characteristics of emotional experience that operate at the individual level, such as individual differences in temperament and personality, as intracultural dimensions. In contrast, characteristics that operate at the cultural level are intercultural dimensions. For example, those from individualistic cultures are more likely than those from collectivistic cultures to report wanting to maximize experiences of positive affect and minimize experiences of negative affect [21]. Members of interdependent cultures also tend to rate negative emotions as less harmful than do those in individualistic contexts. Cross-cultural differences in the way certain emotions are viewed can result in differences in the actual frequency and degree of emotional experiences across cultures. For example, individuals from independent cultural contexts where positive emotions are more desirable to experience than negative emotions, report experiencing positive emotions more frequently than negative emotions because they are considered to be more favorable within that cultural context [22].

One reason why members of different cultures may experience certain emotions to different extents is through a mechanism called situational selection. Harmon-Jones, Harmon-Jones and Summerell note that individuals differ in how positively or negatively they judge different discrete emotions [23]. They posit that an individual with an extremely negative attitude toward anger may engage in emotional situational selection, or the deliberate avoidance of situations that are likely to cause them anger, and thereby experience less anger than someone who holds a more neutral attitude toward the emotion. Likewise, if a *culture* strongly favors (or disapproves of) a particular emotional state then the individual members of the culture may engage in emotional situation selection to change the odds of experiencing the emotion. In this way, entire cultures may engage in situational selection, resulting in patterns of cross-cultural differences in the frequency and degree of experiencing certain discrete emotions. Similarly, in a cross-cultural study of values and emotion, Tamir and colleagues found that participants reported wanting to feel more of the specific emotions that corresponded with the values they endorsed [24]. For example, participants who endorsed the value of self-enhancement (a classically individualistic value) reported a stronger desire to feel the value-consistent emotions of anger and pride. These findings suggest that values precede and shape emotional experience, and that insofar as cultural context shapes individual values, it can also affect the desirability of certain emotions.

People can experience a wide variety emotions, but Diener, Smith, and Fujita suggest that all possible emotions experienced fall under one of six discrete emotion categories [25]. They suggest love, joy, fear, anger, shame, and sadness capture the complete range of human emotion. Diener and colleagues derived these six categories from cognitive [26,27], biological/evolutionary [28,29], and empirical [30,31] perspectives. For the current study, we will consider the emotions of affection, happiness, anxiety and worry, irritation/anger, shame and embarrassment, and sadness, closely mirroring Diener et al.’s six main emotional categories.

### Emotional outcomes of support provision

Inagaki and Orehek suggest that as long as two boundary conditions are met, the provision of SS can be an inherently rewarding experience [7]. First, support must be given freely. That is, support must be given without coercion via interpersonal or societal pressures. Second, providers must believe that the support they are providing is effective. According to Inagaki and Orehek, when these two conditions are met, providers can experience emotional and physiological benefits similar to those enjoyed by the recipients of responsive SS.

When considering whether cross-cultural differences in SS receipt can be used to inform expectations for SS providers, at least two possibilities emerge. The first is that cultural differences in provision will tend to mirror those of receipt. Culturally inappropriate or culturally non-normative styles of support may impose more stress upon providers, leading to worse emotional outcomes. Providing styles of SS deemed inappropriate within a provider’s culture may also negatively influence providers’ sense of the effectiveness of SS provision, leading to further negative emotions. The second possibility is that the cultural factors that shape the dynamics of SS receipt are not generally mirrored in the dynamics of SS provision, and instead operate through different structures. The present study seeks to explore whether the interaction of provider culture and SS type parallels cultural differences in SS receipt.

### The present study

The present study examines the degree to which cross-cultural differences in SS provision mirror cross-cultural differences in SS receipt. Namely, we examine whether SS providers report more discrete negative emotions (anxiety, anger, shame, and sadness) and less discrete positive emotions (affection and happiness) at times when they provided forms of SS that prior research has generally identified as less culturally appropriate. The current study therefore examines differences in the patterns of SS provision and reports of specific emotions among college students from Singapore and the United States. If the dynamics of SS provision parallel those of SS receipt, it would be expected that participants in Singapore would provide more informational support, while participants in the United States would provide more emotional support. Similarly, participants in Singapore would be expected to provide unsolicited support while participants in the United States would be more likely to provide solicited support.

In addition to examining differences in the characteristics of SS provision, we also tested whether the emotional correlates of SS provision varied by culture. If the emotional outcomes of providers parallel those of recipients’ emotions, it would be expected that Singaporean participants would report more affection and happiness and less anxiety, anger, shame, and sadness when providing unsolicited SS and more informational SS. Likewise, we expected that American participants would report more positive and less negative emotions when they provided more solicited support and more emotional support. In addition to these main effects tests of social support characteristics on emotion, we also examined two way interactions between culture and social support solicitation, and between culture and the extent of informational/emotional SS provision. We also compared the emotional correlates of solicited and unsolicited emotional and informational SS. Finally, we examined three-way interactions testing whether cultural differences in emotional or informational SS provision were similar for solicited and unsolicited SS provision. Our reasoning for studying three-way interactions has to do with the nature of unsolicited SS provision. Because unsolicited SS is, by definition, provided without the recipient’s asking, it is much more likely to meet the first of Inagaki and Orehek’s first condition for mutually beneficial SS provision; that support must be given freely [7]. Without the pressure to acquiesce to recipient’s requests for support, unsolicited SS is, by its very nature, given voluntarily. For this reason, we anticipated that the potential for “dysfunctional” SS provision—that is, SS provision that is associated with lower ratings of positive emotions and greater ratings of negative emotions—might be greater for instances of solicited SS.

## Method

### Participants

Responses from 167 University students recruited from Psychology subject pools in the United States (52.7%) and Singapore (47.3%) received research credit for participation in this study. The sample was 73.1% women, and the gender ratio was similar in the United States and Singaporean sample. Mean age was 21.77 (*SD* = 2.77). The Singaporean sample was drawn from an urban private University, while the U.S. sample was drawn from a mid-sized public regional university in a small city in the Pacific Northwest. Despite these differences, there were no statistically significant differences in income or age between the two locations, though there was more variability in age in the U.S. sample. Although 179 University undergraduates participated in this study, 167 produced viable data for analyses. Because this study focused on the qualities of SS provision, we were unable to use data from the ten participants who did not report providing any SS. In addition, one participant was omitted because data collection errors made it impossible to match the responses provided on the two days of the study. Another participant only partially completed the study.

This research was approved by the institutional review boards of Singapore Management University (IRB17-007-A001-117) and Western Washington University (secondary review). Written consent to participate was obtained from all participants.

### Procedure

All participants took part in two different hour-long sessions in campus computer labs in Singapore and the United States. Participants took part on either a Tuesday/Wednesday or a Saturday/Sunday. Participants provided informed consent and used the Qualtrics research platform to respond to questions. Following Kahneman, Krueger, Schkade, Schwarz, & Stone’s Day Reconstruction Method (DRM) procedure [32], all participants were asked to think of their previous day as a series of episodes, and complete a diary sheet listing all episodes that occurred. Participants noted the start and stop times of each episode, listed some descriptive features of the episode, and indicated whether they provided or received social support during the episode. After completing the diary sheet, participants notified a research assistant to help them initiate the Qualtrics questions related to social support provision and receipt. Next, participants responded to questions describing qualities of each episode from the prior day. In addition to the measures described in the measures section, participants indicated their main activities and social interactions for each episode, identified features of SS provision and receipt, and rated several emotions during that episode. SS was defined for participants as being networks of shared social relationships involving reciprocal caring and communication. In addition to examples of emotional and instrumental SS, participants were told that “Sometimes indirect ways of supporting another, such as keeping track of another person’s situation or spending time with the other person are also forms of social support.” Participants answered specific questions about the SS they provided, including the type of recipient of the SS (e.g. friend) who was subsequently referred to as the participant’s “SS partner.” Participants reported an average of 16.04 episodes over the two days. Social support provision was reported in 735 total episodes by 167 different participants (*M* = 4.34; *SD* = 2.62).

### Measures

Descriptive statistics for the variables described in the sections that follow are shown in Table 1, together with the intraclass correlation coefficient, where appropriate.

**Table 1:** Means and standard deviations of SS characteristics and emotions.

	Mean	Standard Deviation	Level 1 N /Level 2 N	ICC
Percent of episodes SS was provided	25%	--	2832/178	--
Informational SS	5.13	1.46	723/166	.339
Emotional SS	4.90	1.90	723/166	.409
Percent of provided SS that was requested	27%	--	735/167	--
Affection	3.08	2.03	2829/178	.344
Happiness	4.63	1.69	2830/178	.271
Irritation/Anger	1.73	1.29	2830/178	.262
Anxiety	2.11	1.59	2829/178	.349
Worry	2.12	1.59	2828/178	.292
Embarrassment	1.35	0.93	2830/178	.336
Shame	1.28	0.97	2830/178	.372
Sadness	1.68	1.21	2830/178	.347

*Note*. Level 1 N = total number of episodes in which variable was recorded; Level 2 N = number of participants who reported variable; ICC = Intraclass correlation coefficient.

#### Emotional SS

This 3-item scale adapted from Maisel and Gable [33] asked participants to describe the extent to which they used emotional SS during the episode in which they had reported providing SS. Participants responded using a scale from 1 (*not at all*) to 7 (*very much*) to indicate the extent to which they tried to provide emotional SS to their episode partner (*M* = 5.13, *SD* = 1.45). Items from this scale include *I tried to offer comforting and encouraging words*, *I tried to tell my partner how much I care about them*, and *I tried to understand my partner*. Cronbach’s alpha was .80 for participants from Singapore and .75 for participants from the United States, and the overall distribution was negatively skewed.

#### Informational SS

This 2-item scale asked participants to describe the extent to which their social support provision during that episode involved providing informational SS. Participants responded using a scale from 1 (*not at all*) to 7 (*very much*) to indicate the extent to which they engaged in each informational social support behavior (*M* = 4.90, *SD* = 1.89). Items from this scale include *I tried to give specific suggestions about how to solve the problem* and *I provided my partner with advice to help them deal with the problem*. Cronbach’s alpha was .95 for participants from Singapore and .96 for participants from the United States. The overall distribution was somewhat negatively skewed.

#### SS request

This measure assessed whether episodes of social support provided by the participant had been requested by the social support recipient. Participants were asked to respond with *Yes* (coded 1) or *No* (coded 0) to the question *Did the other person ask for support*?

#### Emotions

At the start of each episode participants reported the extent to which they had experienced 13 distinct emotions during the episode, using a scale from 1 (*not at all*) to 7 (*very much*). Only the eight measures that aligned with the emotion typology described by Diener, Smith, and Fujita were considered as part of this study [25]. Specifically, we evaluated the extent to which culture and SS characteristics predicted affection, happiness, irritation/anger, anxiety, worry, embarrassment, shame, and sadness. Note that this list of emotions has been previously used in cross-cultural research using the Day Reconstruction Method [32]. Because the distribution of each negatively valanced emotion was positively skewed, a natural logarithm was calculated to help reduce the effect of extreme negative emotions scores on the analyses. All tests of negative emotions were conducted both using the original metric and the log-transformed variables. The two positively valanced variables were not severely skewed, and no transformations were used.

## Results

### Cultural differences in the frequency and characteristics of SS provision

Overall, participants in the U.S. sample reported more episodes (*M* = 16.93, *sd* = 5.31) than did those in Singapore (*M* = 15.05, *sd* = 4.54), *t*(165) = 2.67, *p* = .015. However, those in Singapore reported providing SS to others in a greater percentage of their episodes, *t*(165) = -3.69, *p* < .001 (Singapore mean percentage = 33.04, *sd* = 18.08; U.S. *M* = 24.11, *sd* = 12.34). There were no cultural differences in the percent of SS provision episodes that participants described as having been requested, *t(*165) = 1.81, *p* = .073. Singapore’s mean person-level percentage of requested SS provision reports was 24.10 (*sd* = 27.96), while the mean percentage in the United States was 32.47 (*sd* = 31.61).

Two multilevel modeling analyses were used to test whether culture predicted differences in the continuous variables of informational and emotional SS provision. Location did not predict the amount of reported emotional SS provision (*b* = -.12; *t*(164) = -0.67, *p* = .505) or informational SS provision (*b* = -.18; *t*(164) = -0.88, *p* = .380).

### Emotional correlates of SS provision

#### Data analysis overview

All analyses predicting discrete emotions were conducted using multilevel modeling to account for the nested data structure. Specifically, variables associated with specific episodes (including emotions and all social support provision characteristics) were analyzed at Level 1, while characteristics of the individual (i.e., culture) were analyzed at Level 2. All Level 1 variables were group mean centered prior to analyses. For consistency, random effects for each variable were initially tested and were included in all subsequent analyses if the random effect was statistically significant, using *p* < .10. Analyses considering emotional SS provision and informational SS provision were conducted separately for each emotion, yielding the 16 different combinations of SS type and emotional outcome (affection, happiness, irritation/anger, anxiety, worry, embarrassment, shame, and sadness).

The large number of complex analyses that were conducted as part of this study raises concerns about capitalization on chance. For *descriptive* purposes, Tables 2 and 3 provide an indication of regression coefficients that are statistically significant at *p* < .01, *p* < .05, and *p* < .10. These values are useful for understanding patterns observed across multiple variables and may be of interest to the reader. However, because of the large number of analyses we only interpret results that are statistically significant at *p* < .01. For parsimony, most of the regression coefficients and standard errors are presented only in the Tables, and the bulk of this section interprets the results without repeating the values that were not statistically significant.

**Table 2:** Culture and emotional correlates of emotional social support provision.

	Affection		Happiness		Anger /Irritation		Anxiety		Worry		Embarrass-ment		Shame		Sadness	
Predictor	b value	SE	b value	SE	b value	SE	b value	SE	b value	SE	b value	SE	b value	SE	b value	SE
Intercept	4.00	.18	5.40	.11	.24	.04	.49	.06	.96	.03	.78	.02	.15	.03	.88	.03
Location	.50*	.26	-.24	.17	.12†	.06	-.09	.07	.10*	.05	.00	.03	.04	.05	.04	.04
Emotion SS	.62**	.12	.35**	.10	-.07**	.03	-.07†	.04	-.01	.02	-.02†	.01	-.00	.02	.01	.01
Requested	-.25	.21	-.25†	.14	.13*	.07	.03	.06	.04	.04	.02	.03	-.01	.03	-.02	.04
Emotion SS x Requested	-.03	.21	.04	.18	.11*	.05	.10	.07	.05	.05	-.02	.02	.05	.04	-.02	.03
Emotion SS x Location	-.12	.18	-.04	.13	.03	.04	.01	.05	-.06	.04	.00	.01	-.04†	.02	-.00	.02
Requested x Location	.04	.31	-.05	.25	-.05	.10	.10	.07	.09	.07	.01	.04	-.01	.05	.04	.05
Emotion SS x Location x Requested	.34	.30	.13	.25	-.25**	.07	-.09	.09	.03	.07	-.02	.03	-.07	.06	-.04	.05

*Note*. Robust standard errors are used for all analyses. All negative emotions (anger, anxiety, worry, embarrassment, shame, and sadness) were logarithmically transformed prior to calculations.

**p < .01

* p < .05

† *p* < .10. Underlined values indicate that models included random Level 2 variability for the underlined variables. All intercept coefficients included statistically significant random variability at *p* < .01.

**Table 3:** Culture and emotional correlates of informational social support provision.

	Affection			Happiness		Anger /Irritation		Anxiety			Worry		Embarrass-ment		Shame		Sadness	
Predictor		b value	SE	b value	SE	b value	SE	b value	SE	b value		SE	b value	SE	b value	SE	b value	SE
Intercept		3.99	.18	5.41	.11	.25	.04	.49	.06	.96		.03	.78	.02	.15	.03	.88	.03
Location		.54*	.25	-.24	.17	.12†	.06	-.08	.08	.10*		.05	.00	.03	.05	.05	.04	.04
Information SS		-.05	.08	.07	.07	-.01	.02	-.00	.02	.00		.02	-.01	.01	.00	.01	.02*	.01
Requested		-.20	.23	-.25†	.14	.11†	.06	.02	.06	.04		.04	.01	.03	-.00	.04	-.03	.04
Information SS x Requested		.14	.17	-.00	.11	.11*	.03	.03	.05	-.01		.03	.03†	.02	-.01	.03	-.00	.02
Information SS x Location		.02	.12	-.05	.08	.05*	.02	.02	.05	.01		.03	.01	.01	.00	.02	-.01	.01
Requested x Location		-.10	.35	-.06	.26	-.01	.10	.12	.09	.08		.07	.03	.04	.00	.05	.05	.06
Information SS x Location x Requested		-.04	.26	-.10	.21	-.19**	.07	-.14*	.07	-.00		.06	-.08**	.02	-.07	.05	-.02	.04

*Note*. Robust standard errors are used for all analyses. All negative emotions (anger, anxiety, worry, embarrassment, shame, and sadness) were logarithmically transformed prior to calculations.

**p < .01

* p < .05

† *p* < .10. Underlined values indicate that models included random Level 2 variability for the underlined variables. All intercept coefficients included statistically significant random variability at *p* < .01.

The formulas below summarize the main analyses that test the main effects of social support provision (informational or emotional), whether that support was requested, and location (Singapore or United States), as well as the two way interactions between SS provision and SS request, between location by SS provision, and between location by SS request, as well as the three-way interaction between location, SS provision, and SS request. Decisions about whether Level 1 predictor variables should be modeled as fixed or random effects were determined through preliminary analyses looking only at the Level 1 variables.

$$Level\;1:\;Emotion_{ij}=\pi_{0j}+\pi_{1j}(Social\;Support)+\pi_{2j}(Request)+\pi_{3j}(Social\;Support\;x\;Request)+e_{ij}$$

$$Level\;2:\;\pi_{0j}=\beta_{00}+\beta_{01}(Location)+r_{0j}$$

$$\pi_{0j}=\beta_{10}+\beta_{11}(Location)+r_{1j}$$

$$\pi_{0j}=\beta_{20}+\beta_{21}(Location)+r_{2j}$$

$$\pi_{0j}=\beta_{30}+\beta_{31}(Location)+r_{3j}$$

In the Level 1 formula above, emotion_ij_, is person j’s specific emotion at time i. That score was predicted by *π*_*0*j_, the person-level intercept for that emotion, by *π*_*1*j_, which is the effect of SS provision (emotional or informational) for person j, by *π*_*2*j_, the person-level effect of SS request, by *π*_*3*j_, which is the effect of and the centered social support by request interaction, and by *e*_*ij*_ which is error for person j at time i. Variability at Level 2 is captured by estimates of the intercept (*β*_*00*_), the average of each level 1 effect across participants (*β*_*10*_ through *β*_*30*_; i.e., *β*_*10*_ is the cross-person average slope of SS provision on emotion), the effect of location on the intercept(*β*_*01*_), interactions between location and SS provision and SS request (*β*_*11*_ and *β*_*21*_), and the three-way interaction (*β*_*31*_), as well as error (*r*_*0j*_ through *r*_*3j*_). Note that the *r*_*0j*_ error term indicates individual variability in the intercept, while *r*_*1j*_ through *r*_*3j*_ capture between-person differences in the magnitude of the corresponding slope; these random factors were included only when preliminary analyses indicated between-person variability at *p* < .10 (as shown by the underlined values in the Tables).

The results of these analyses are summarized in Table 2 for emotional SS analyses and Table 3 for informational SS. Analyses were conducted using the log-transformed negative emotion variables. Unless otherwise noted, all results are similar when conducted without the logarithmic transformation of the dependent variable. To aid in interpretability, estimated values in the Figures were calculated using non-transformed variables.

In addition a set of supplementary analyses tested whether the results remained consistent if the type of recipient (family member, friend, romantic partner, or acquaintance) of SS was statistically considered. Cultural differences in the target of the SS provision were considered as a possible alternative explanation for the observed cultural differences in social support. This step was important because even though most SS was provided to friends or to romantic partners in both samples, preliminary analyses indicated that there were cultural differences in the targets of the SS. Specifically, those in Singapore were relatively more likely to provide SS to family members, while those in the United States were especially likely to provide SS to acquaintances. Tests were conducted by using a set of three dummy coded variables to indicate whether the participant reported providing support to friends (the reference category), family members, romantic partners, or acquaintances. The dummy coded covariates were used to predict the *π*_*0j*_ at level 2 in the formulas above. Because statistically considering SS recipient did not alter the effects described below and reported in Tables 2 and 3, these tests are not presented in this manuscript. Details on these analyses are available upon request.

#### Cultural differences in the effects of emotional SS provision and solicitation

Emotional SS provision predicted participant ratings of greater affection (*b* = .62, *p* < .001) and happiness (*b* = .35, *p* < .001), as well as less irritation/anger (*b* = -.06, *p* = .006). Unique relationships between emotional SS provision and anxiety, worry, and embarrassment were not statistically significant in these multivariate models. There was no indication that emotional SS provision predicted sadness or shame. Likewise, with the exception of anger, which needs to be interpreted in the context of a 3-way interaction, tests of SS request indicated that emotions differed based on whether or not the social support had been requested by the recipient, at *p* < .01. Further, there was no suggestion that discrete emotional correlates of emotional SS provision differed based on whether that support was or was not solicited, as evidenced by the non-significant interactions between emotional SS provision and whether or not SS was requested.

**Fig 1:**
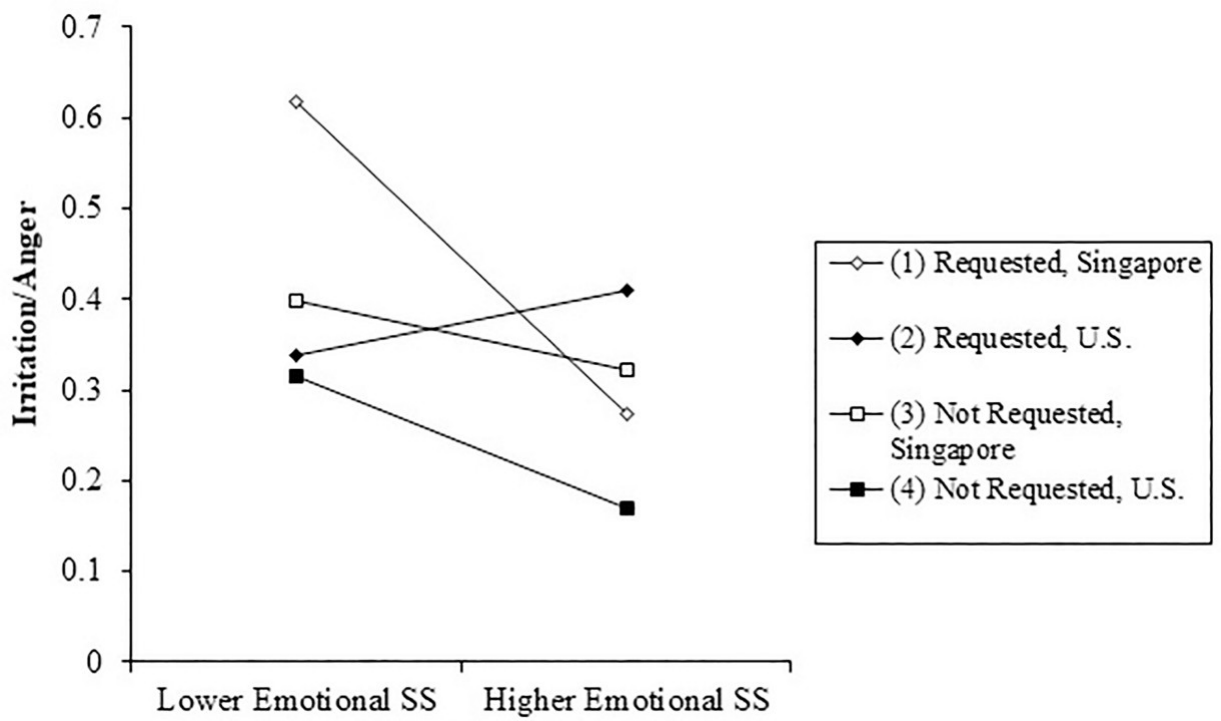
Interaction among culture, emotional SS provision, and solicitation of SS on irritation.

For irritation/anger only, the main effect of emotional SS provision and request (as well as their interaction) should be interpreted in the context of a 3-way interaction between culture, emotional SS provision, and whether the SS was solicited by the recipient (*b* = -.25, *p* = .002). As shown in Fig 1, more provision of emotional SS predicted less irritation/anger overall. However, at times when SS was requested, the emotional consequences of emotional SS provision varied by culture. For participants from Singapore, reports of irritation/anger were highest when participants reported few attempts to provide solicited emotional SS and irritation was lowest with more emotional SS provision (simple slope *b* = -.49, *p* < .001). In contrast, for those in the U.S. sample, requested emotional SS provision did not predict irritation/anger (simple slope *b* = -.10, *p* = .362). Although this cross-over interaction pattern was not evident for unsolicited SS, unsolicited emotional SS provision was associated with relatively less anger both in Singapore (simple slope *b* = -.13, *p* < .042) and in the U.S. sample (simple slope *b* = -.14, *p* = .018). Likewise, there were no other main effects or two-way interactions related to SS request and culture on other emotions. Although the 3-way interaction pattern was not observed for the effects of emotional SS provision on any emotion other than irritation/anger, as described below similar results were obtained in tests of the emotional correlates of informational SS provision.

#### Cultural differences in the effects of informational SS provision and solicitation

**Fig 2:**
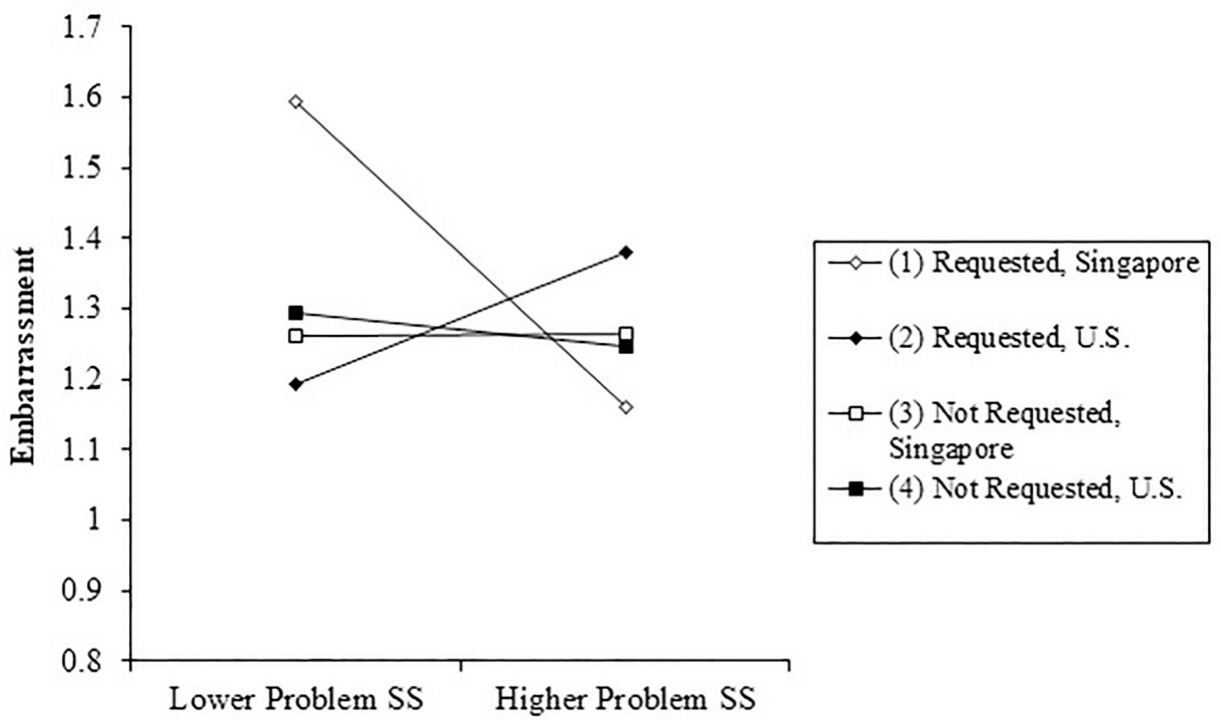
Interaction among culture, informational SS provision, and solicitation of SS on embarrassment.

A summary of the analyses of the provision of informational SS, as shown in Table 3, shows few consistent direct effects of culture, the provision of informational SS, or requests for SS on any of the emotional outcomes. However, a similar 3-way interaction to the one described above was observed for anger (*b* = -.19, *p* = .010) and embarrassment (*b* = -.08, *p* = .001), and a similar trend existed for anxiety (*b* = -.14, *p* = .039). Each of these effects was also statistically significant for tests of the emotional outcome without a logarithmic transformation. Although Fig 2 only shows the pattern of estimated effects for embarrassment, the direction of the effects is similar for irritation/anger. Specifically, in both cultural contexts, there was no link between unsolicited informational support provision and negative emotions. However, when support was requested, the emotional correlates (i.e., embarrassment and irritation) of informational SS provision varied by culture. In the Singapore sample, relatively more provision of solicited informational SS predicted less embarrassment (and less irritation), while in the U.S. sample more provision of solicited informational SS predicted more embarrassment and irritation.

## Discussion

Our primary interest in this study was observing whether cultural differences in SS provision would follow similar patterns to those previously observed within SS receipt, or if patterns for SS providers would differ from the findings in previous research on SS recipients.

If SS provision processes paralleled SS receipt processes, participants in Singapore would be expected to provide more informational support and more unsolicited support to others, and participants in the United States would provide more emotional support and more solicited support to others. Likewise, participants in Singapore would be expected to report more affection and happiness and less anxiety, anger/irritation, worry, embarrassment, shame, and sadness when providing informational and unsolicited support, and participants in the United States would report similar emotions in instances in which they provided emotional and solicited SS.

Overall, the patterns of cross-cultural differences in SS provision of the present study do not mirror cross-cultural differences in patterns of SS receipt, and raise the possibility that cross-cultural differences in emotion responses to SS provision operate differently than for SS receipt. Rather than expecting provider and recipient dynamics to mirror each other, equity theory considers SS to be an equitable exchange, where each member of the dyad brings their own dynamics to the exchange, meaning the emotions and SS behaviors that each member of the dyad experiences during the interaction do not need to be exactly the same [34]. In the context of SS, this means that patterns of SS provision and patterns of SS receipt do not need to perfectly match for a SS exchange to be successful. Similarly, SS providers and SS recipients do not need to experience the same emotions for a SS exchange to be successful. The findings from this study support the idea that even within the same SS exchange, providers and recipients may have different emotional outcomes, and this may influence the perceived success of the SS exchange.

When we examined only episodes in which SS was provided, our findings did not adhere to our expectations drawn from previous research. First, although the direction of the difference between solicited and unsolicited SS was anticipated, the proportion of support that was requested did not differ significantly between Singapore and the United States. Secondly, the amount of informational support provision and the amount of emotional SS provision did not differ between Singapore and the United States. These results differ from previous research that has suggested that SS exchange in interdependent cultures such as Singapore tends to emphasize problem-focused SS, such as informational SS, whereas SS exchange in more independent cultures such as the United States tends to emphasize emotional SS.

Our findings suggested that there were some notable cultural differences in patterns of SS provision. First, participants in Singapore provided SS to others overall in more episodes than participants in the United States. This is in keeping with previous findings regarding SS exchange in more interdependent cultures. Though most SS was provided to either friends or to romantic partners in both Singapore and the United States, individuals in Singapore were relatively more likely to report providing SS to family members, while individuals in the United States were relatively more likely to provide SS to acquaintances. This is likely due to the fact that many college students in Singapore live with their families, while this is not necessarily the case in the United States. The Singaporean participants may have had more exposure to family members than the American participants, allowing them more opportunities to provide SS to family. Similarly, many college students in the United States have jobs or engage in multiple extracurricular activities outside of school, whereas this is less common in Singapore. Participants in the United States may interact with acquaintances on a more regular basis than participants in Singapore, allowing them more opportunities to provide SS to acquaintances.

Our findings indicate that regardless of cultural context, when providing emotional SS, participants reported greater affection and happiness, as well as less irritation/anger and anxiety. Fostering happiness and affection and lessening negative emotions are key characteristics of emotional SS. Therefore, it makes sense that providing emotional support would be related to greater happiness and affection. If emotional SS is genuinely expressed, it is not surprising that providers experienced greater positive emotions and less negative emotions. After all, research on SS suggests that emotion-focused SS provision results in more positive outcomes than problem-focused SS provision [6].

However, regardless of cultural context, greater informational SS provision did not predict any of the emotional outcomes. It is possible that there is more contextual variability when it comes to providing informational SS, particularly if these emotional outcomes are not being considered in a cross-cultural context. This could be due to the fact that informational SS can be used to address a wide range of problems, from providing advice on how to resolve a conflict with a romantic partner to information on how to best prepare for a presentation at school or work. This means that informational support might include small, easy acts of providing information, or large acts that require a great amount of time or resources on the part of the provider. Informational support therefore can require varied levels of provider investment, likely involving different emotional outcomes. The positively and negatively valenced emotional outcomes for providers of informational SS may balance out across situations and people, resulting in null overall effects.

When considering culture, specific emotions, SS requests, and SS provision together, the results indicated an interesting interactive relationship. Overall, episodes with more emotional SS were characterized by more affection and happiness and less irritation/anger and anxiety. However, when considering episodes in which emotional SS was requested, only ratings of irritation/anger varied significantly by culture. In Singapore, ratings of irritation/anger were lowest when participants reported more provision of solicited emotional SS and greatest when participants reported less provision of solicited emotional SS. In contrast, in the United States, irritation/anger was lower with less provision of solicited emotional SS and highest with more provision of solicited emotional SS. It could be that participants in Singapore who provided relatively more emotional SS in response to their partners requests did so because the requests seemed important. Therefore, there would not be a reason to feel particularly irritated/angry. However, if in Singapore, a SS partner requested emotional SS that the participants did not consider to be necessary or appropriate, the provider would not provide as much support, and might feel anger/irritation at the request. In contrast, within the U.S. sample, participants may have felt irritated/angry when asked to provide more emotional SS. This pattern makes sense within the context of what we know about cultural differences in SS. The goal of emotional SS is reassurance and esteem-building, but in an interdependent cultural context, this type of support can violate cultural expectations of modesty and appropriate emotional expression [35]. In addition, work on cultural differences in the importance of saving face suggests that negative emotions may arise when group members break face, meaning that they fail to appropriately follow norms related to hierarchy and societal expectations [36]. Losing face can be considered selfish, rude, and inappropriate, as it causes discomfort for all who are involved in the social interaction [37]. All of these factors could result in SS providers in Singapore feeling angry/irritated at being requested to provide a form of SS that both members of the SS exchange would recognize as being culturally inappropriate. However, an exception could be made in the instance of a request for emotional SS that seemed especially important, because knowing the inappropriateness of a request for emotional SS would probably make a SS recipient less likely to request it unless they felt that emotional SS was truly needed.

Requests for emotional SS in the U.S. context may have made participants feel overburdened, and may have made the provision feel less voluntary, violating Inagaki and Orehek’s first criterion for beneficial SS provision: support must be given freely [7]. This pattern did not hold true for *unsolicited* emotional SS, further supporting the idea that providing inappropriate or burdensome forms of SS would lead SS providers to experience more irritation/anger.

Similarly, provision of unsolicited SS was not associated with negative emotions for providers in either culture, whereas informational SS that *was* requested was associated with negative emotions. This association between solicited informational SS provision and negative emotions varied by culture. In Singapore, greater provision of solicited informational SS was related to less irritation/anger and embarrassment. In the United States greater provision of solicited informational SS was related to more irritation/anger, anxiety, and embarrassment. Again, culture moderates the association between solicited SS provision (in this case informational SS provision) and worse emotional outcomes for providers. For both cultures, unsolicited SS provision did not carry the same costs to providers as solicited support provision. Alternatively, participants in Singapore may have felt fewer negative emotions when providing informational SS because informational support is a more culturally appropriate form of support. Participants in the United States may have experienced more irritation/anger and embarrassment when providing informational SS because they provided a less culturally appropriate type of support to their partners.

Rather than expecting identical input and emotional outcomes for providers and recipients, equity theory posits that both parties should perceive the exchange of resources as being equal [38]. If a provider feels as if the SS receiver is requesting too much support, or requiring more of the provider than they are willing to give, the exchange is not equal and can have negative outcomes for the provider. This concern is less prevalent when SS is provided without the recipient requesting it, as there is less potential for the provider to feel coerced by their partner’s request.

The current results may differ from previous SS research because our measure of SS provision assessed the extent to which providers believed they had provided each type of SS. It is possible that what providers tend to categorize as a particular type of SS (informational vs. emotional or solicited vs. unsolicited) could be categorized differently by recipients. In this way, studying providers might have yielded seemingly different patterns of SS than have been observed in the largely recipient-focused literature. For these reasons, our findings did not neatly parallel those of the SS receipt studies. Alternatively, these findings may suggest that cultural differences are most apparent when SS exchanges are seen as inequitable or otherwise dysfunctional. Tolstoy noted of families, “All happy families are alike; each unhappy family is unhappy in its own way.” Similarly, cultural differences may be most subtle when SS exchange is most beneficial and most apparent when SS exchange is dysfunctional. Making one’s partner feel loved and supported may evoke the same positive emotions universally, whereas cultures may have unique patterns of response to unsuccessful or unfavorable SS exchanges.

### Limitations and future directions

One potential limitation to this study is that participants were asked to self-report their experiences from only two previous days regardless of whether or not they had provided SS that day. Though Kahneman et al.’s Day Reconstruction Method [32] is an accepted procedure for collecting retrospective accounts of previous days, it is not without its limitations. Participants misremembering or intentionally omitting information is a possible concern with any self-report study. In addition, although participants reported a large number of episodes, only an average of four episodes per person involved the provision of SS, thereby reducing statistical power, especially for tests of cross-level interactions. Future research might expand upon this study by obtaining more episodes involving SS provision and by asking participants to provide a brief explanation of the context in which SS was provided. The rich contextual information may allow a more complete understanding of potential cross-cultural differences in emotional responses to SS provision and the Day Reconstruction Method would be a useful tool for capturing this kind of information. Likewise, analyses involving more episodes of SS provision would provide a more powerful replication of these results; the results reported here should be considered tentative, pending replication.

Future studies exploring cross-cultural SS should build upon this research by providing participants with a wider variety of SS options to report on. In the current study, we used a 2-item measure of problem-focused SS that focused exclusively on informational SS. Both questions regarding problem-focused SS only involved giving advice or providing suggestions to fix the problem, and failed to include other types of problem-focused SS such as instrumental assistance, like giving someone a ride or buying them groceries. It is possible that potential cultural differences were not observed because participants were not asked about instances of instrumental problem-focused SS. Future research should be sure to include a variety of measures regarding different types of SS in order to more thoroughly capture nuances in responses. Likewise, although the multi-item SS provision measures were reliable in each cultural context, we did not use multilevel approaches to ascertain cultural measurement invariance, and it is possible that factor structures may vary by culture [39].

One final potential limitation to this study comes from the sample characteristics. The study was designed to examine cross-cultural differences in SS provision and emotional outcomes among a sample of individuals living in a independent context and a sample of individuals living in an interdependent context. However, Singapore may not be an ideal example of an interdependent culture. Singapore is a relatively young, highly diverse country that has experienced a great deal of Western influence in recent decades [16]. While previous research has indicated that Singapore is higher in interdependence than the United States is [40], Singapore may be comparatively individualistic compared to classically and often studied nations in East Asia like Japan and China. Furthermore, by sampling exclusively from a young, urban, student population, it is plausible that the Singaporeans included in our sample are considerably more individualistic/Westernized than the general population. Compared to the U.S. sample, the Singaporean sample were more likely to live at home with family rather than with friends and roommates. Singaporean participants lived in a very densely populated urban environment versus the American’s suburban or campus living. These differences are not necessarily cultural ones, and—to the extent that they drove differences in SS provision—may have limited our ability to study cross-cultural differences. Because the intent of this study was to focus on personal dynamics in the process of SS provision, culture was considered a backdrop of this study rather than a focus. For this reason, this study did not include a specific measure of interdependent and independent cultural values, potentially limiting the generalizability of these results. Because of these factors, future research should replicate and extend the study of SS provision in different interdependent contexts to better capture the cultural variability between highly independent cultures and highly interdependent cultures, as well as include specific measure of cultural values to help pinpoint the cultural components that may be contributing to these findings.

### Implications and conclusions

Overall, this study highlights both cross-cultural differences and cross-cultural similarities in the emotional effects of SS for providers. Although much of the existing SS research has focused on the recipient, this study focuses on the experience of the SS provider. The findings from this study support the idea that it is extremely important to consider culture when studying SS processes. A deeper understanding of the role culture plays in SS interactions has a number of real-world implications. Due to globalization, the increasing ease of travel, and social media, engaging in regular cross-cultural interactions is easier and more common than it has ever been before. Because of this, individuals around the world are able to make connections and maintain relationships with people who exist in cultural contexts that are completely different from their own. The global expansion of social networks calls for an increased understanding of the mechanisms behind cross-cultural interactions. Similarly, people are more mobile, and many individuals have the option to relocate to a country they were not born in. Integrating into a new culture has numerous implications for stress and well-being and cross-cultural SS may play an important role in mitigating the negative effects of culture shock. Finally, research on cross-cultural SS can help us to better understand the nuances of acculturation in a cross-generational context. Because it is relatively common to relocate, multiculturalism within families where children are growing up in a different cultural context than their parents did, is increasingly normalized. Understanding how individuals from different cultural contexts prefer to provide and receive SS can help facilitate mutually beneficial SS exchanges among loved ones with different cultural values. Overall, these findings highlight the importance of research on cross-cultural SS provision and emphasize the need for more research on SS provision in general.
